# Supplementary material for: Molecular phyloecology suggests a trophic shift concurrent with the evolution of the first birds
Source: Commun Biol. 2021 May 13;4:547. doi: 10.1038/s42003-021-02067-4 (PMC8119460; doi:10.1038/s42003-021-02067-4)
Supplement: Supplementary file 2 — Description of Additional Supplementary Files [file 42003_2021_2067_MOESM2_ESM.pdf]

Description of Additional Supplementary Files

File name: Supplementary Data 1

Description: Supplementary Data 1 Species and GenBank sequences of genes used in this study.

File name: Supplementary Data 2

Description: The genes under relatively relaxed selection ($k<1$) and relatively intensified selection ($k>1$) in ancestral bird.

File name: Supplementary Data 3

Description: The genes involved in digestive system pathways.
