# Supplementary material for: Molecular phyloecology suggests a trophic shift concurrent with the evolution of the first birds
Source: Commun Biol. 2021 May 13;4:547. doi: 10.1038/s42003-021-02067-4 (PMC8119460; doi:10.1038/s42003-021-02067-4)
Supplement: Supplementary file 6 — Reporting Summary [file 42003_2021_2067_MOESM6_ESM.pdf]

Corresponding author(s): Yonghua Wu
Last updated by author(s): 2021/3/20

Reporting Summary

Nature Research wishes to improve the reproducibility of the work that we publish. This form provides structure for consistency and transparency in reporting. For further information on Nature Research policies, see our Editorial Policies and the Editorial Policy Checklist.

Statistics

For all statistical analyses, confirm that the following items are present in the figure legend, table legend, main text, or Methods section.

n/a	Confirmed	
☒	☐	The exact sample size (*n*) for each experimental group/condition, given as a discrete number and unit of measurement
☒	☐	A statement on whether measurements were taken from distinct samples or whether the same sample was measured repeatedly
☒	☐	The statistical test(s) used AND whether they are one- or two-sided<br>*Only common tests should be described solely by name; describe more complex techniques in the Methods section.*
☒	☐	A description of all covariates tested
☒	☐	A description of any assumptions or corrections, such as tests of normality and adjustment for multiple comparisons
☒	☐	A full description of the statistical parameters including central tendency (e.g. means) or other basic estimates (e.g. regression coefficient) AND variation (e.g. standard deviation) or associated estimates of uncertainty (e.g. confidence intervals)
☒	☐	For null hypothesis testing, the test statistic (e.g. *F*, *t*, *r*) with confidence intervals, effect sizes, degrees of freedom and *P* value noted<br>*Give P values as exact values whenever suitable.*
☒	☐	For Bayesian analysis, information on the choice of priors and Markov chain Monte Carlo settings
☒	☐	For hierarchical and complex designs, identification of the appropriate level for tests and full reporting of outcomes
☒	☐	Estimates of effect sizes (e.g. Cohen's *d*, Pearson's *r*), indicating how they were calculated

Our web collection on statistics for biologists contains articles on many of the points above.

Software and code

Policy information about availability of computer code

Data collection	No software was used.
Data analysis	Two published softwares PAML (version 4.9i) and RELAX were used for data analyses in this study.

For manuscripts utilizing custom algorithms or software that are central to the research but not yet described in published literature, software must be made available to editors and reviewers. We strongly encourage code deposition in a community repository (e.g. GitHub). See the Nature Research guidelines for submitting code & software for further information.

Data

Policy information about availability of data

All manuscripts must include a data availability statement. This statement should provide the following information, where applicable:

- Accession codes, unique identifiers, or web links for publicly available datasets
- A list of figures that have associated raw data
- A description of any restrictions on data availability

The transcriptome sequencing data were deposited into the National Center for Biotechnology Information Sequence Read Archive database under accession numbers (SRR12237019-20). All other data needed to evaluate the conclusions in the paper are present in the paper and/or the supplementary materials, or are available from the corresponding author on reasonable request.

Field-specific reporting

Please select the one below that is the best fit for your research. If you are not sure, read the appropriate sections before making your selection.

☐ Life sciences ☐ Behavioural & social sciences ☒ Ecological, evolutionary & environmental sciences

For a reference copy of the document with all sections, see nature.com/documents/nr-reporting-summary-flat.pdf

Ecological, evolutionary & environmental sciences study design

All studies must disclose on these points even when the disclosure is negative.

Study description	We mainly included 95 species in this study, including 73 bird species, representing the majority of living bird orders, and 22 reptile species, including five crocodilians, six turtles, and 11 squamates. With these species, their phylogeny is constructed and the adaptive evolution of 83 digestive system-related genes was analyzed. The evidence of positive and/or intensified selection is used to infer the diet of ancestral animals based on a recently developed molecular phyloecological method.
Research sample	We mainly included 95 species in this study, and these species were used because of the availability of their gene sequences from GenBank database. Two samples, including one ostrich (three months old) and one emu (six months old), were used for transcriptome sequencing in this study. The two individuals are the same individuals used in one of our previous studies (Wu and Wang 2019). The combined gene sequence data of the GenBank species and the two species was used for analyses in this study.
Sampling strategy	The 95 species used in this study largely represent the main groups of living birds and reptiles. And especially, we included 73 bird species representing the majority of living bird orders (36/39). For the 73 bird species included, the majority come from Neognathae, with relatively little species of Palaeognathae. For the Palaeognathae species included, the GenBank sequences of many of our focal genes were missing upon our initial sequence analyses, especially for the ostrich (Struthio camelus) and the emu (Dromaius novaehollandiae), and thus we selected the two species for transcriptome sequencing. Our data cover species from almost all main groups of living birds and reptiles.
Data collection	The tissue samples of our target species were collected in our lab, and these samples were used for transcriptome sequencing using Illumina HiSeq X-ten (Biomarker Technology Co., Beijing). And the assembled coding sequences of our genes were used for data analyses.
Timing and spatial scale	Tissue samples were used for transcriptome sequencing in December 2017 and the coding sequences of our target genes were obtained about half an year later.
Data exclusions	The genes (e.g., amylase genes) with sequences unavailable or available for only few bird species were excluded from our analyses.
Reproducibility	We used two different methods (PAML and RELAX) for our data analyses (diet reconstruction of ancestral birds) and similar results were obtained, strengthening our findings in this study.
Randomization	This is not relevant to this study. The species used in this study represent almost all of main living bird and reptile groups. The species of each group cited from GenBank were originally selected by different researchers to genome sequencing due to different reasons (e.g., economic value and sample availability).
Blinding	Blinding is not relevant to this study because all data was collected and analyzed by the same author (Yonghua Wu).

Did the study involve field work? ☐ Yes ☒ No

Reporting for specific materials, systems and methods

We require information from authors about some types of materials, experimental systems and methods used in many studies. Here, indicate whether each material, system or method listed is relevant to your study. If you are not sure if a list item applies to your research, read the appropriate section before selecting a response.

Materials & experimental systems

n/a	Involved in the study	
☒	☐	Antibodies
☒	☐	Eukaryotic cell lines
☒	☐	Palaeontology and archaeology
☐	☒	Animals and other organisms
☒	☐	Human research participants
☒	☐	Clinical data
☒	☐	Dual use research of concern

Methods

n/a	Involved in the study	
☒	☐	ChIP-seq
☒	☐	Flow cytometry
☒	☐	MRI-based neuroimaging

Animals and other organisms

Policy information about studies involving animals; ARRIVE guidelines recommended for reporting animal research

Laboratory animals	This study did not involve laboratory animals.
Wild animals	One ostrich (three months old) and one emu (six months old) were used for sampling. The two individuals were the same two individuals used in one of our previous studies (Wu and Wang 2019). The two animals were bought from an artificial breeding company (Quanxin, Daqing), and after tissue sampling, the ostrich was selected to making skeleton specimen and the emu was discarded.
Field-collected samples	This study did not include field-collected samples.
Ethics oversight	The experimental procedures were carried out following an animal ethics approval granted by Northeast Normal University. All experimental procedures in this study were approved by the National Animal Research Authority of Northeast Normal University, China (approval number: NENU-20080416), and the Forestry Bureau of Jilin Province of China (approval number: [2006]178).

Note that full information on the approval of the study protocol must also be provided in the manuscript.
